# Supplementary material for: A US case-control study to estimate infant group B streptococcal disease serological thresholds of risk-reduction
Source: Nat Commun. 2025 Oct 23;16:9381. doi: 10.1038/s41467-025-64324-y (PMC12549997; doi:10.1038/s41467-025-64324-y)
Supplement: Supplementary file 1 — Supplementary Info [file 41467_2025_64324_MOESM1_ESM.pdf]

Supplementary Information

Contents

Supplementary Figure 1. Relationship between blood spot storage time before testing and serotype-specific anti-capsular polysaccharide IgG antibody concentration (IA and IB) ... 2

Supplementary Figure 2. Relationship between blood spot storage time before testing and serotype-specific anti-capsular polysaccharide IgG antibody concentration (II and III) ... 3

Supplementary Figure 3. Relationship between blood spot storage time before testing and serotype-specific anti-capsular polysaccharide IgG antibody concentration (IV and V) ... 4

Supplementary Figure 4. Relationship among cases between timing of DBS collection relative to disease onset and serotype-specific log anti-capsular polysaccharide IgG antibody concentration ... 5

Supplementary Figure 5. Relationship among cases between timing of DBS collection relative to disease onset and log anti-capsular polysaccharide IgG antibody concentrations ... 6

Supplementary Figure 6. Receiver Operator Characteristic (ROC) curves for early-onset disease and late-onset disease ... 7

Supplementary Figure 7. Risk curves for anti-capsular polysaccharide IgG with additional sensitivity analysis for IAP ... 8

Supplementary Figure 8. Risk curves for anti-capsular polysaccharide IgG antibody concentrations including race/ethnicity as covariates ... 9

Supplementary Figure 9. Comparing threshold point estimates from CALM to the Bayesian Absolute Disease Rate (ADR) and Weighted Logistic Regression (WLR) methods used in Madhi, S. et al NEJM 2023 ... 10

Supplementary Figure 10. Log anti-capsular polysaccharide IgG antibody concentrations among controls for key variables ... 11

Supplementary Figure 11. Directed Acyclic Graph ... 13

Supplementary Table 1. Wilcoxon p-values and Areas Under the Curve by Serotype and Age at Onset ... 14

Supplementary Table 2. Sample size stratified by serotype, age at onset, and anti-capsular polysaccharide IgG antibody concentrations (above or below the lower limit of quantification) ... 15

Supplementary Table 3. Comparing point estimates from CALM to the Bayesian Absolute Disease Rate (ADR) and Weighted Logistic Regression (WLR) methods used in Madhi, S. et al NEJM 2023 ... 16

Supplementary Table 4. This study was reviewed and approved by the following non-CDC Institutional Review Boards (IRBs) ... 17

Supplementary Methods Appendix A. CALM Methodology ... 18

1. Introduction ... 18

2. Confounding Effects on Dose-Response Curves ... 18

Supplementary Figure 12. The absolute and relative covariate-specific dose response curve ... 19

3. Scaled Logit Model (SLM) ... 19

4. Covariate Adjusted Logit Model (CALM) ... 20

Supplementary Methods Appendix B. Population disease rate calculations ... 22

Supplementary References ... 23

Supplementary Figure 1. Relationship between blood spot storage time before testing and serotype-specific anti-capsular polysaccharide IgG antibody concentration (IA and IB)

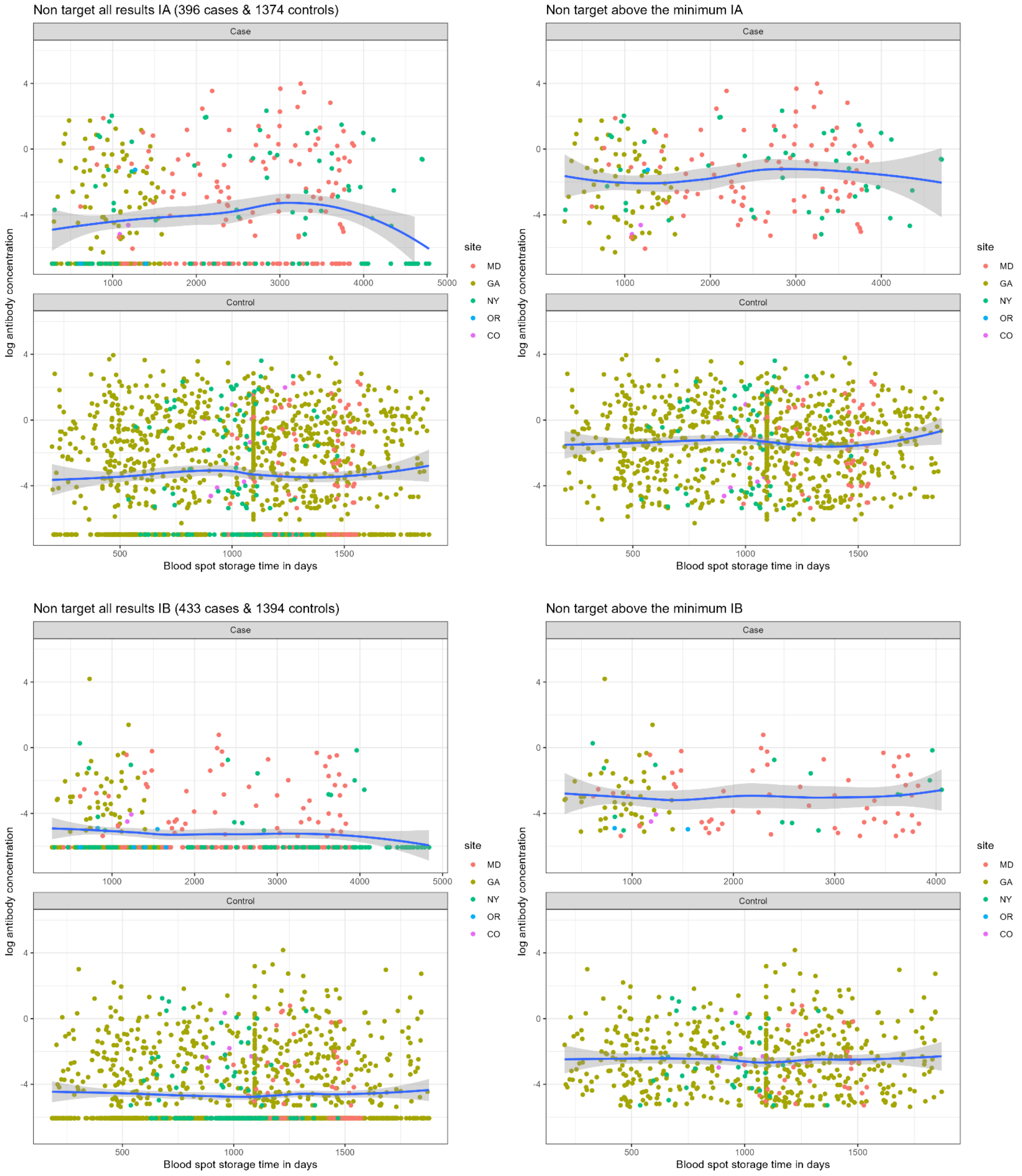

Legend: Serotype-specific plots showing the relationship between blood spot storage time (days) and log anti-GBS CPS IgG antibody concentration (mcg/mL) for IA and IB. The assessment was restricted to sites that stored blood spots in refrigeration, as there were more concerns regarding stability under refrigeration compared to freezing conditions. Colors indicate the study site (MD = Maryland, GA = Georgia, NY = New York, OR = Oregon, CO = Colorado). Results are shown for non-target serotypes (e.g. for the IA plots, anti-IA CPS IgG levels are shown for cases and controls classified as serotype IB, II, III, IV, or V).

Supplementary Figure 2. Relationship between blood spot storage time before testing and serotype-specific anti-capsular polysaccharide IgG antibody concentration (II and III)

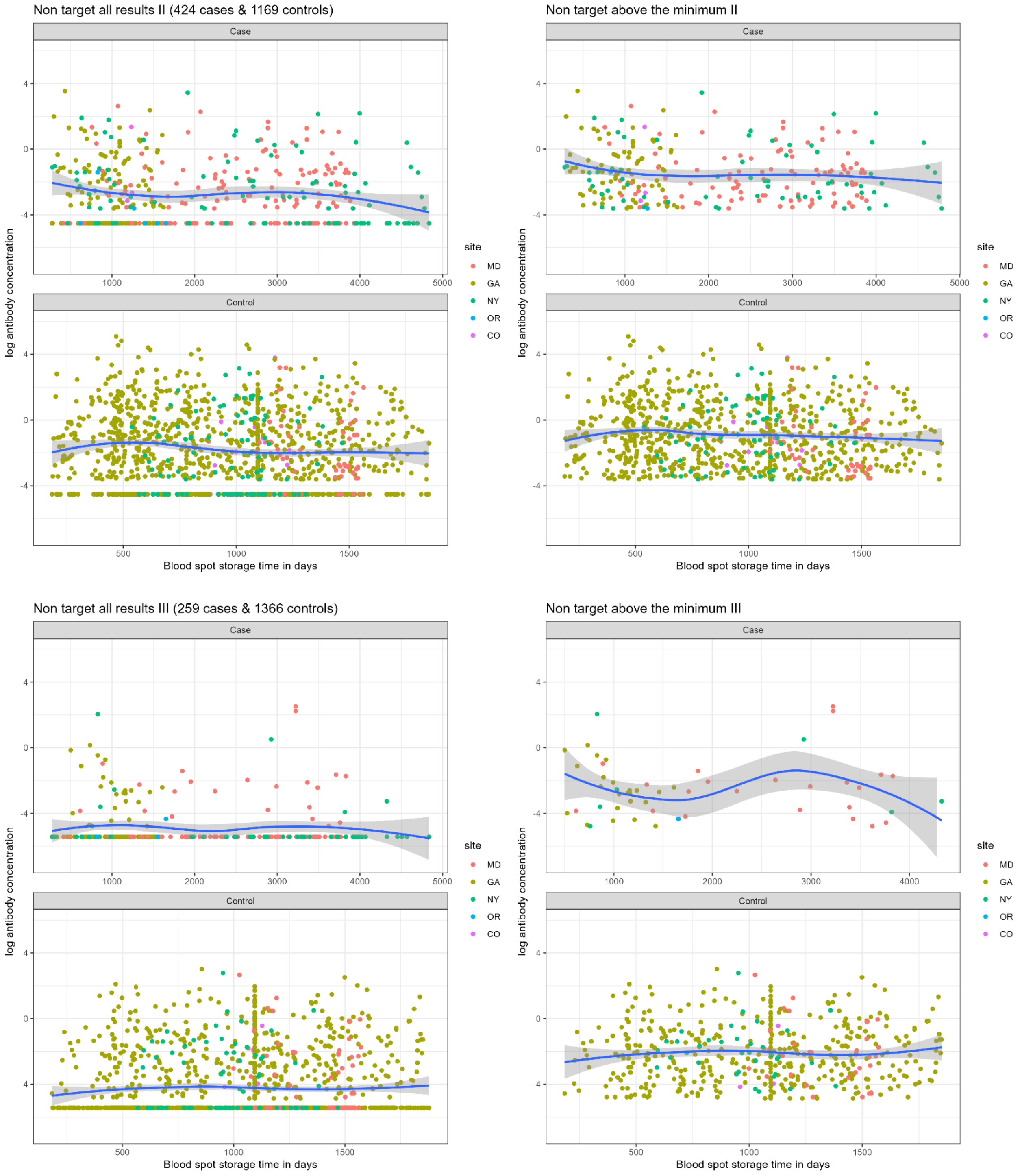

Legend: Serotype-specific plots showing the relationship between blood spot storage time (days) and log anti-GBS CPS IgG antibody concentration (mcg/mL) for II and III. The assessment was restricted to sites that stored blood spots in refrigeration, as there were more concerns regarding stability under refrigeration compared to freezing conditions. Colors indicate the study site (MD = Maryland, GA = Georgia, NY = New York, OR = Oregon, CO = Colorado). Results are shown for non-target serotypes (e.g. for the II plots, anti-II CPS IgG levels are shown for cases and controls classified as serotype IA, IB, III, IV, or V).

Supplementary Figure 3. Relationship between blood spot storage time before testing and serotype-specific anti-capsular polysaccharide IgG antibody concentration (IV and V)

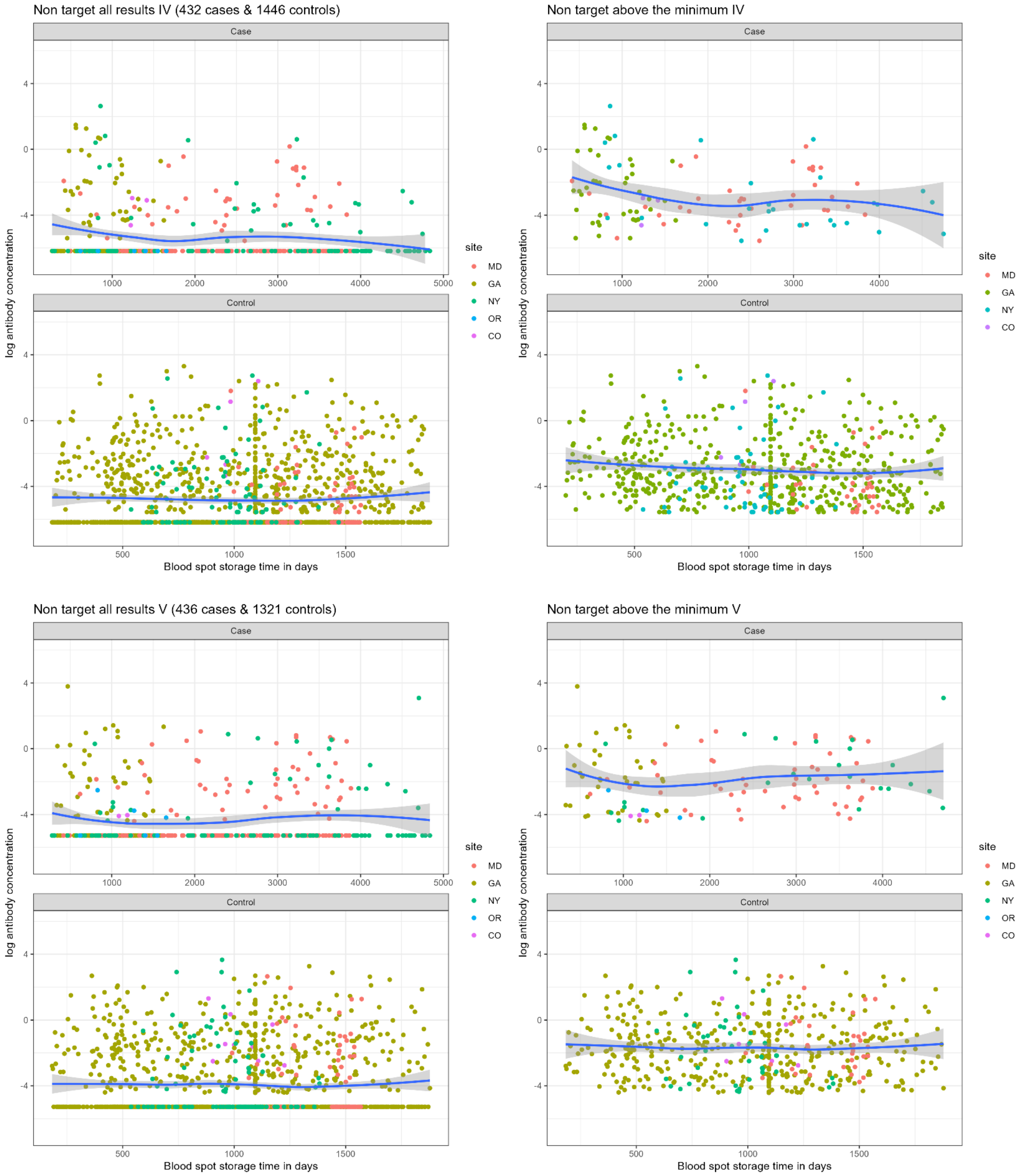

Legend: Serotype-specific plots showing the relationship between blood spot storage time (days) and log anti-GBS CPS IgG antibody concentration (mcg/mL) for IV and V. The assessment was restricted to sites that stored blood spots in refrigeration, as there were more concerns regarding stability under refrigeration compared to freezing conditions. Colors indicate the study site (MD = Maryland, GA = Georgia, NY = New York, OR = Oregon, CO = Colorado). Results are shown for non-target serotypes (e.g. for the IV plots, anti-II CPS IgG levels are shown for cases and controls classified as serotype IA, IB, II, III, or V).

Supplementary Figure 4. Relationship among cases between timing of DBS collection relative to disease onset and serotype-specific log anti-capsular polysaccharide IgG antibody concentration

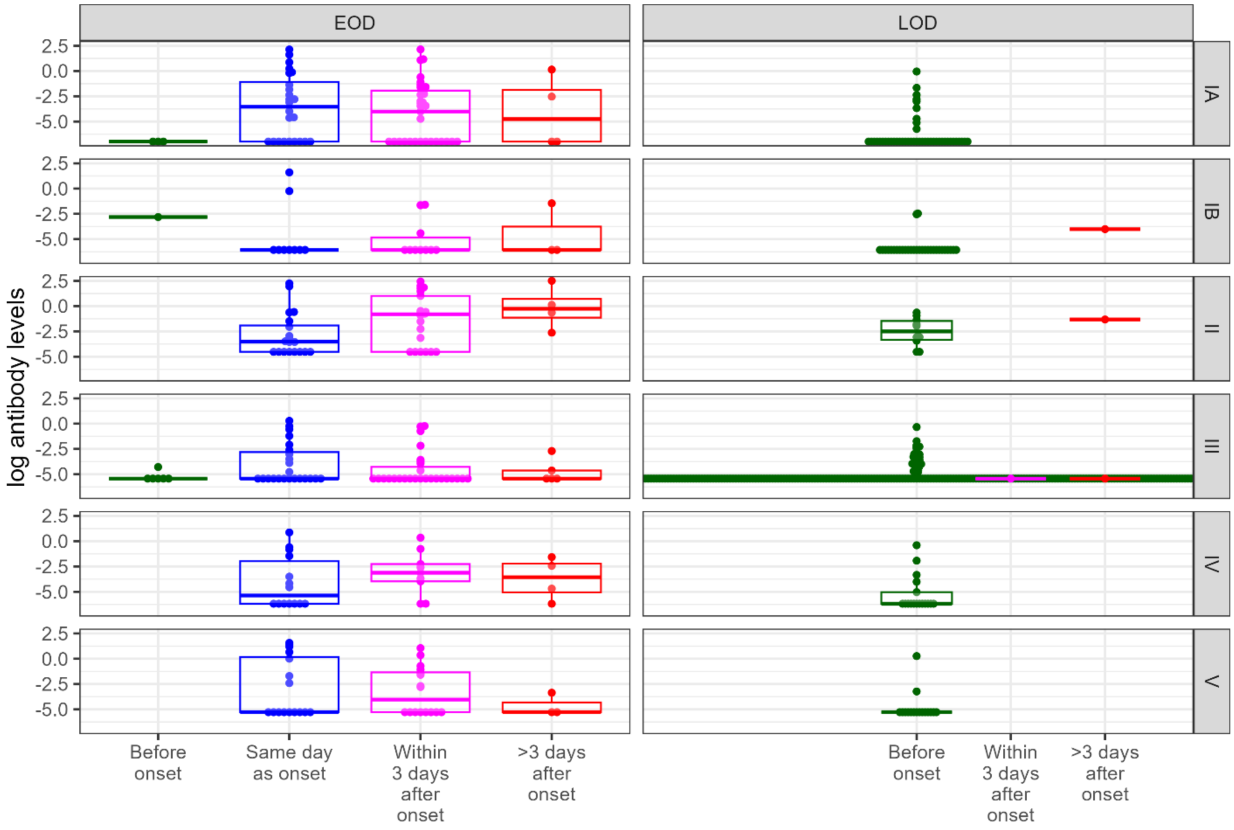

Legend: Log antibody concentrations for early-onset disease (EOD) and late-onset disease (LOD) cases by serotype (rows), color coded by relationship between timing of dried blood spot (DBS) collection relative to disease onset.

Supplementary Figure 5. Relationship among cases between timing of DBS collection relative to disease onset and log anti-capsular polysaccharide IgG antibody concentrations

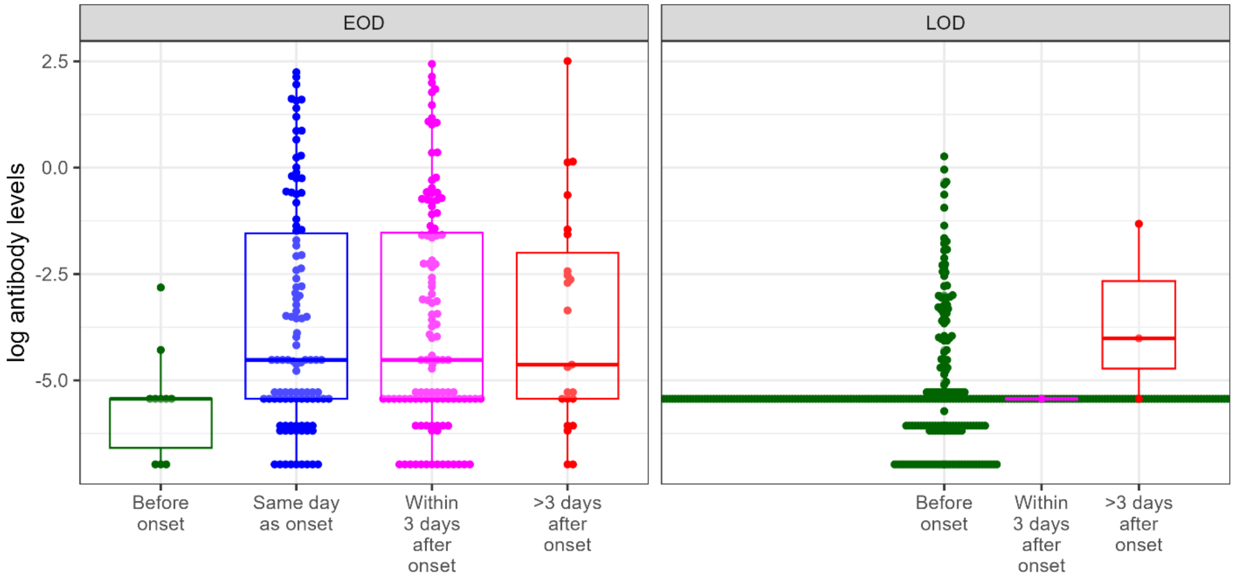

Legend: Log antibody concentrations for early-onset disease (EOD) and late-onset disease (LOD) cases, color coded by relationship between timing of dried blood spot (DBS) collection relative to disease onset.

Supplementary Figure 6. Receiver Operator Characteristic (ROC) curves for early-onset disease and late-onset disease

<u>Early-onset disease</u>

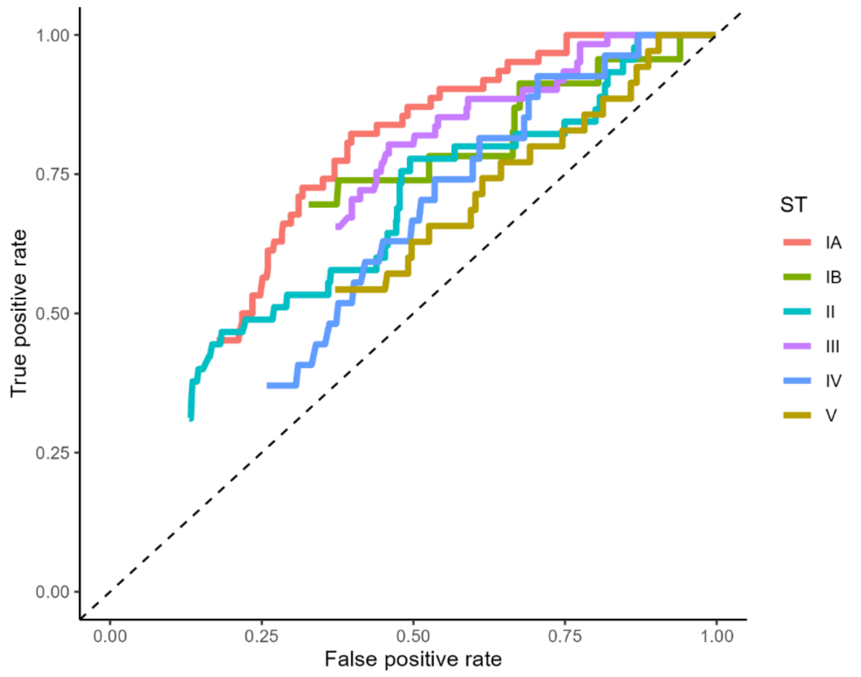

<u>Late-onset disease</u>

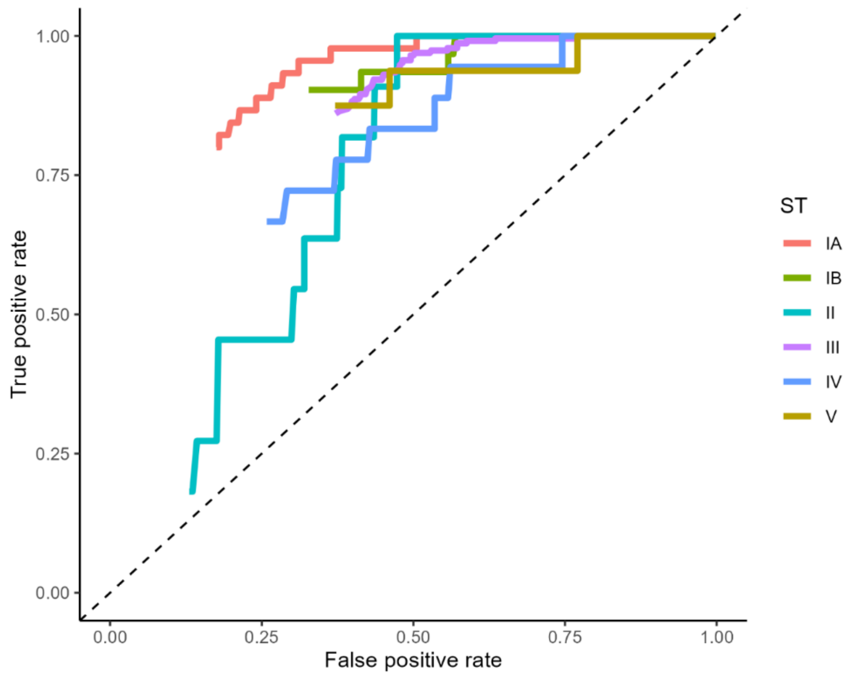

Legend: Receiver operator characteristic (ROC) curves for early-onset (top row) and late-onset (bottom row) disease by serotype (ST) (colors).

Supplementary Figure 7. Risk curves for anti-capsular polysaccharide IgG with additional sensitivity analysis for IAP

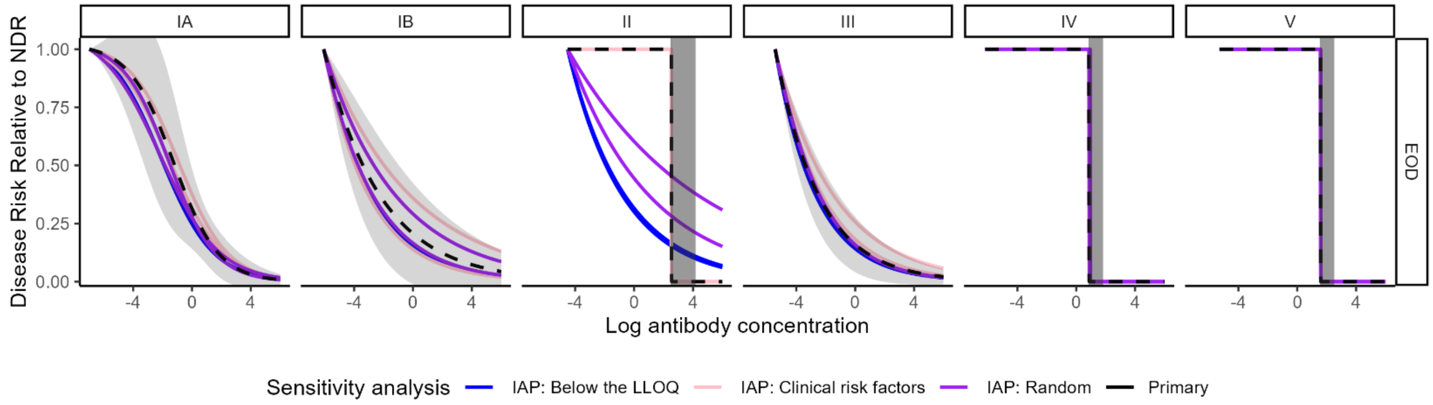

Legend:

This figure provides an additional sensitivity analysis to that shown in Figure 6 (middle panel). “Below the LLOQ” curves (blue) were generated after a small number* of randomly selected controls who received 2 or more hours of IAP and had antibody concentrations below the LLOQ were treated as counterfactual cases (i.e., reclassified as cases for analytic purposes). “Clinical risk factors” curves (pink) were generated after a small number of randomly selected controls who received 2 or more hours of IAP and had clinical risk factors highly associated with EOD were treated as counterfactual cases. “Random” curves (purple) were generated after a small number of randomly selected controls who received 2 or more hours of IAP were treated as counterfactual cases. “Below the LLOQ” curves,“ Clinical risk factors” curves, and “Random” curves were generated using the CALM method and repeated twice to allow for variability in the random selections.

*2-5 controls for each serotype were treated as counterfactual cases with the exact number determined by applying the rate of cases among colonized women in the absence of IAP: 11 per 1000 live births.

IAP = intrapartum antibiotic prophylaxis, LLOQ = lower limit of quantification

Supplementary Figure 8. Risk curves for anti-capsular polysaccharide IgG antibody concentrations including race/ethnicity as covariates

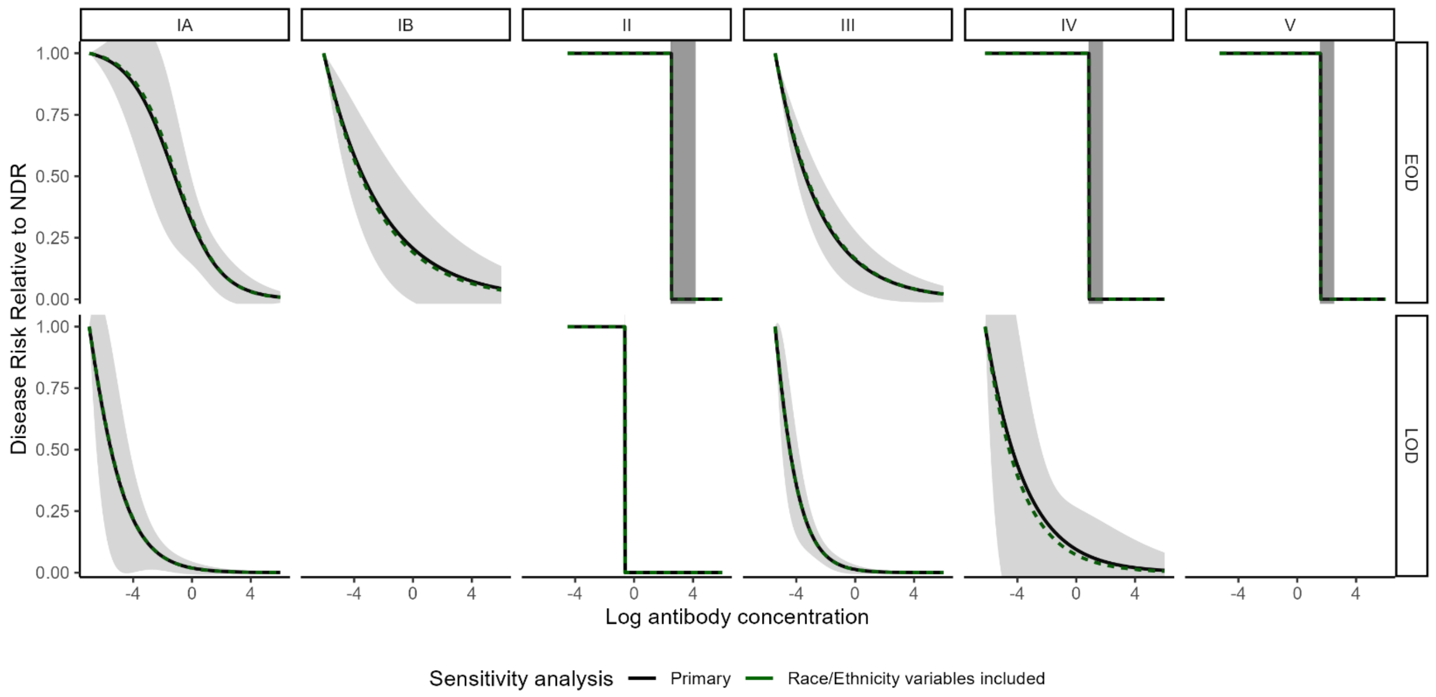

Legend: Risk curves for the primary analysis were generated using the covariate adjusted logit model (CALM), adjusted for gestational age, intraamniotic infection, and study site and are shown for early-onset disease (EOD) (top row) and late-onset disease (LOD) (bottom row) by serotype (columns). Risk curves for the sensitivity analysis including race were generated using the same methods, with race categorized as: Non-Hispanic Black, Non-Hispanic white, or other.

Supplementary Figure 9. Comparing threshold point estimates from CALM to the Bayesian Absolute Disease Rate (ADR) and Weighted Logistic Regression (WLR) methods used in Madhi, S. et al NEJM 2023

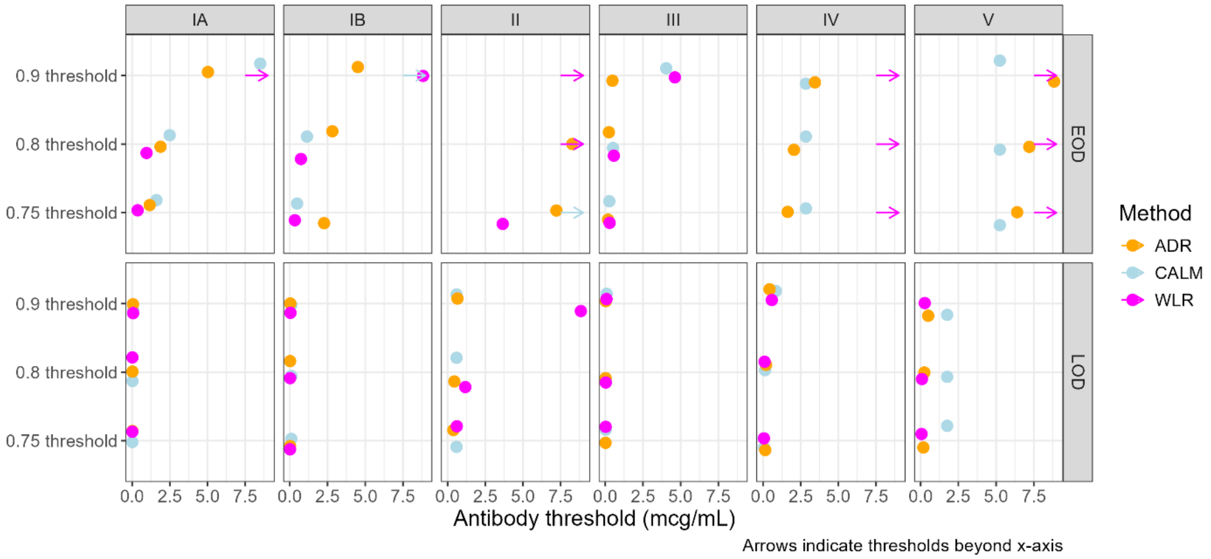

Legend: 0.75, 0.8, and 0.9 thresholds for early-onset disease (EOD) and late-onset disease (LOD) by serotype (columns) using three different methods:

ADR = Bayesian Absolute Disease Rate method used in Madhi, S. et al. NEJM 2023

CALM = Covariate Adjusted Logit Model

WLR = Weighted Logistic Regression method used in Madhi, S. et al. NEJM 2023

Supplementary Figure 10. Log anti-capsular polysaccharide IgG antibody concentrations among controls for key variables

Study site antibody distribution among controls

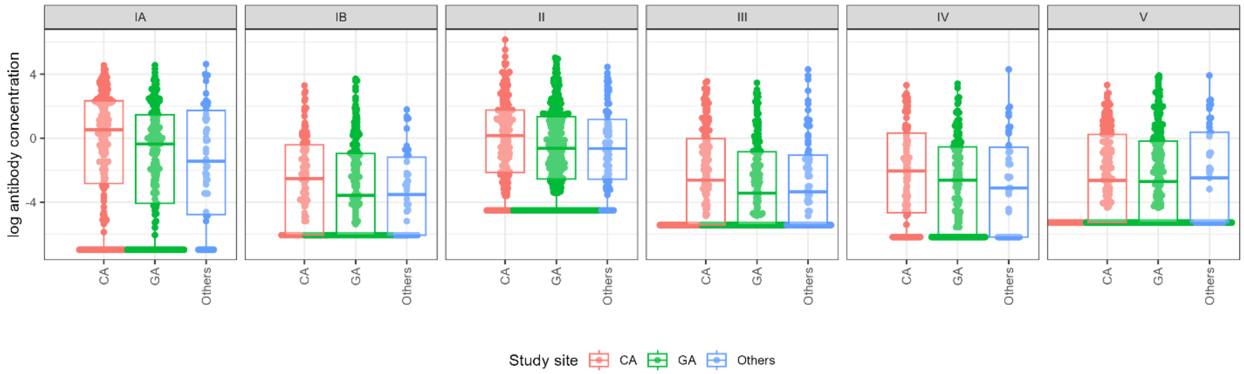

Intraamniotic infection antibody distribution among controls

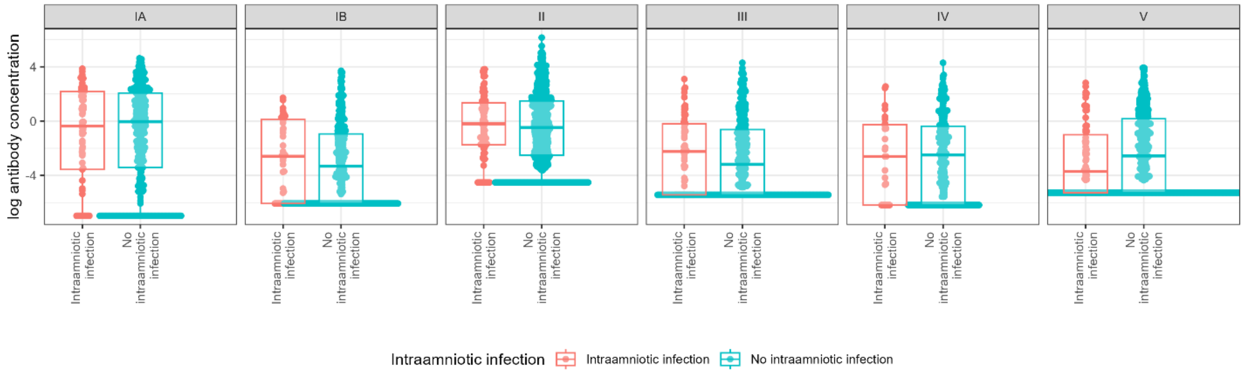

IAP antibody distribution among controls

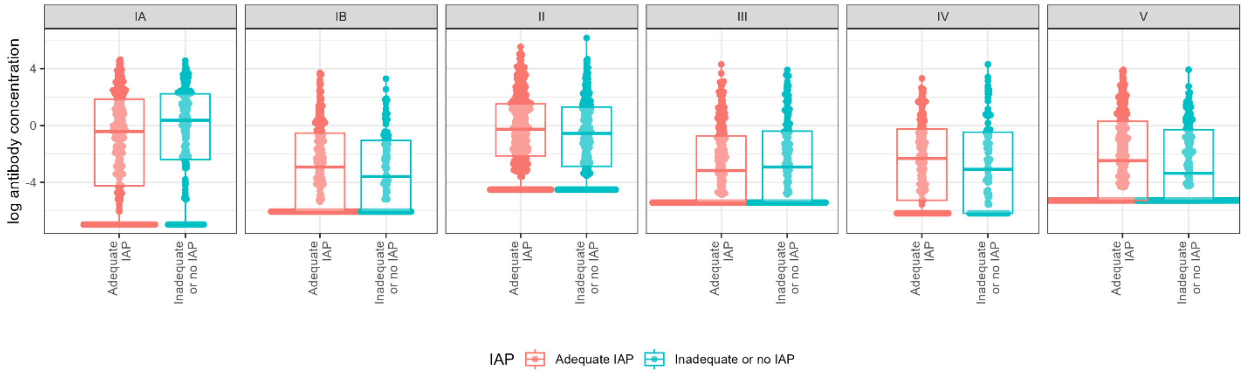

Binary gestational age antibody distribution among controls

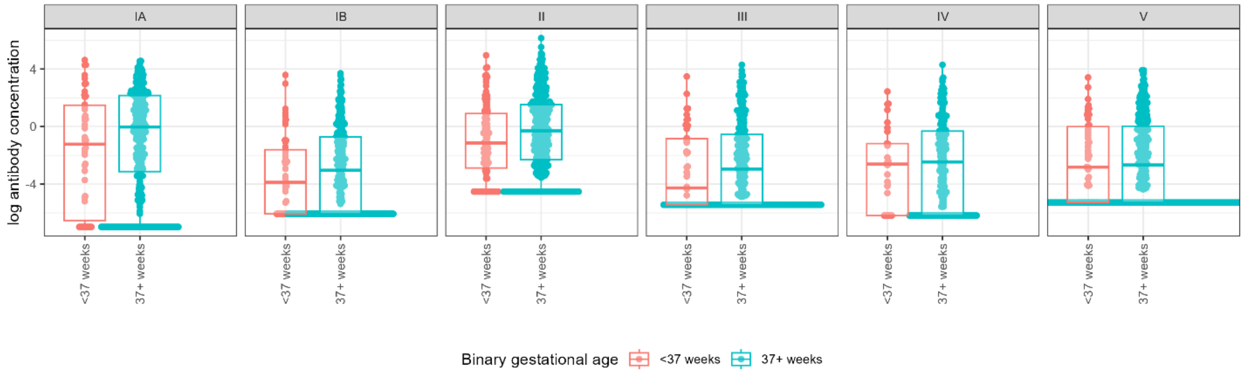

Legend: Examining the relationship between log anti-capsular polysaccharide IgG antibody concentrations (mcg/mL) and key covariate, including study site, presence or absence of intraamniotic infection, adequate vs. inadequate or no IAP, gestational age less than 37 weeks or greater than or equal to 37 weeks, among controls and by serotype (columns); CA = California; GA = Georgia; IAP = Intrapartum antibiotic prophylaxis

Supplementary Figure 11. Directed Acyclic Graph

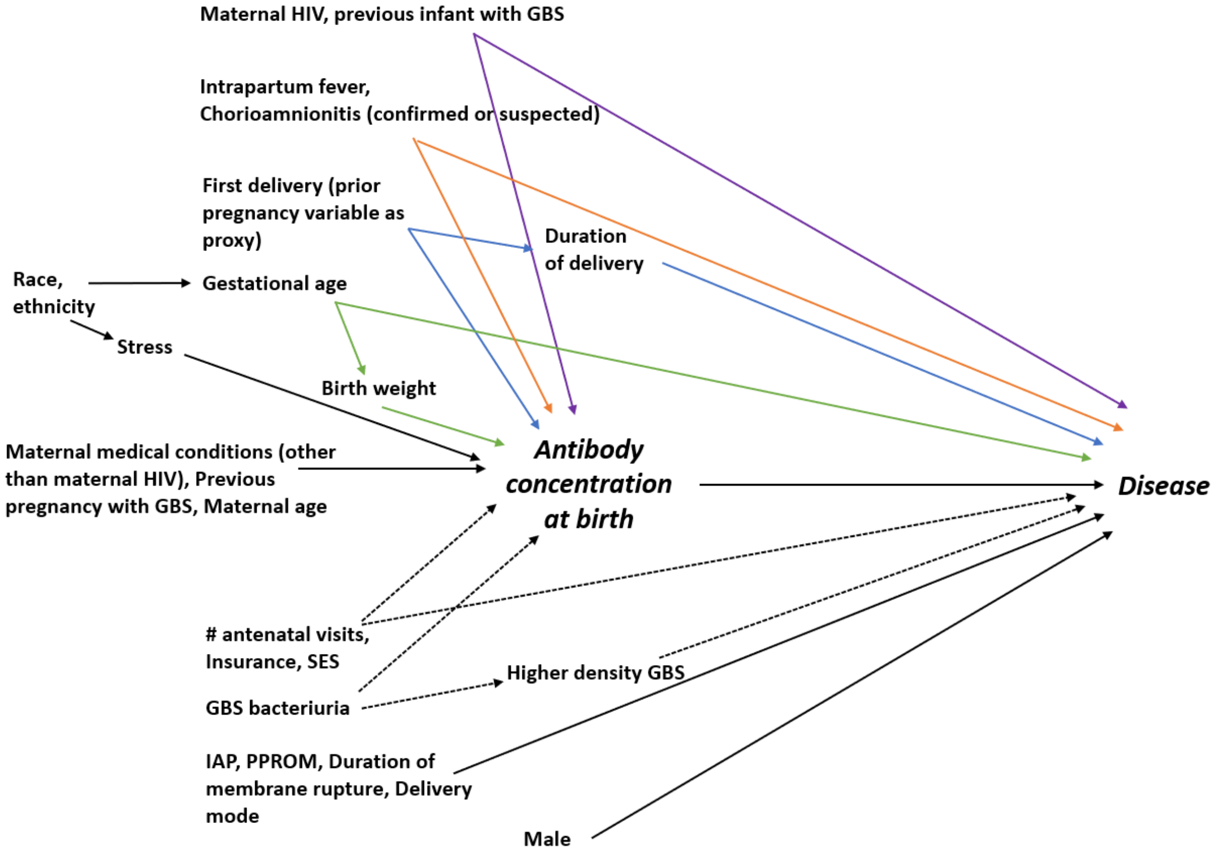

Legend: Directed acyclic graph to describe the relationship between variables and identify potential confounders. Dotted lines indicate uncertain associations. Colored lines indicate confounders.

Supplementary Table 1. Wilcoxon p-values and Areas Under the Curve by Serotype and Age at Onset

Disease	Serotype	Wilcoxon two-sided p-value	AUCs (95% bounds)
EOD	IA	<0.001	0.75 (0.69-0.79)
EOD	IB	0.003	0.68 (0.57-0.78)
EOD	II	<0.001	0.66 (0.57-0.73)
EOD	III	<0.001	0.68 (0.63-0.74)
EOD	IV	0.05	0.61 (0.48-0.71)
EOD	V	0.08	0.59 (0.47-0.68)
LOD	IA	<0.001	0.87 (0.84-0.90)
LOD	IB	<0.001	0.80 (0.76-0.85)
LOD	II	0.007	0.74 (0.65-0.83)
LOD	III	<0.001	0.77 (0.75-0.80)
LOD	IV	<0.001	0.75 (0.65-0.83)
LOD	V	<0.001	0.76 (0.68-0.83)

EOD = Early-onset disease; LOD = Late-onset disease

Supplementary Table 2. Sample size stratified by serotype, age at onset, and anti-capsular polysaccharide IgG antibody concentrations (above or below the lower limit of quantification)

	EOD above LLOQ	EOD below LLOQ	LOD above LLOQ	LOD below LLOQ	Control above LLOQ	Control below LLOQ
IA	34 (55%)	28	9 (20%)	36	390 (82%)	83
IB	7 (30%)	16	3 (10%)	28	234 (67%)	114
II	31 (69%)	14	9 (82%)	2	614 (87%)	92
III	21 (34%)	40	32 (14%)	198	278 (63%)	165
IV	17 (63%)	10	6 (33%)	12	201 (74%)	70
V	16 (46%)	19	2 (13%)	14	329 (63%)	194

EOD = Early-onset disease; LOD = Late-onset disease. LLOQ = lower limit of quantification

Note: this is the sample used for CALM curve generation so excludes those missing key covariates

Supplementary Table 3. Comparing point estimates from CALM to the Bayesian Absolute Disease Rate (ADR) and Weighted Logistic Regression (WLR) methods used in Madhi, S. et al NEJM 2023

Serotype	Disease	Method	0.75 threshold	0.8 threshold	0.9 threshold
IA	EOD	ADR	1.16	1.88	5.03
IA	EOD	CALM	1.61	2.49	8.49
IA	EOD	WLR	0.37	0.96	18.91
IA	LOD	ADR	0.02	0.02	0.05
IA	LOD	CALM	0.01	0.02	0.07
IA	LOD	WLR	0.01	0.02	0.07
IB	EOD	ADR	2.27	2.82	4.51
IB	EOD	CALM	0.48	1.13	16.34
IB	EOD	WLR	0.33	0.74	8.83
IB	LOD	ADR	0.01	0.02	0.03
IB	LOD	CALM	0.1*	0.1*	0.1*
IB	LOD	WLR	0.01	0.02	0.04
II	EOD	ADR	7.22	8.27	9.74
II	EOD	CALM	12.91*	12.91*	12.91*
II	EOD	WLR	3.66	9.34	171.16
II	LOD	ADR	0.38	0.45	0.66
II	LOD	CALM	0.59*	0.59*	0.59*
II	LOD	WLR	0.61	1.18	8.84
III	EOD	ADR	0.18	0.24	0.48
III	EOD	CALM	0.27	0.52	4.04
III	EOD	WLR	0.29	0.57	4.62
III	LOD	ADR	0.02	0.02	0.03
III	LOD	CALM	0.03	0.04	0.09
III	LOD	WLR	0.03	0.03	0.08
IV	EOD	ADR	1.64	2.05	3.45
IV	EOD	CALM	2.85*	2.85*	2.85*
IV	EOD	WLR	24.7	111.99	12265.24
IV	LOD	ADR	0.15	0.21	0.43
IV	LOD	CALM	0.08	0.14	0.85
IV	LOD	WLR	0.06	0.11	0.59
V	EOD	ADR	6.4	7.2	8.85
V	EOD	CALM	5.25*	5.25*	5.25*
V	EOD	WLR	999.81	7110.6	3150805
V	LOD	ADR	0.18	0.24	0.5
V	LOD	CALM	1.76*	1.76*	1.76*
V	LOD	WLR	0.06	0.08	0.27

* zero risk protective threshold used

Supplementary Table 4. This study was reviewed and approved by the following non-CDC Institutional Review Boards (IRBs)

**IRBs**
California Health & Human Services Agency
Kaiser Foundation Hospitals
Colorado Department of Public Health and Environment IRB
Colorado Multiple IRB
Connecticut Department of Public Health Human Investigations Committee
Yale University IRB
Georgia Department of Public Health IRB
Emory University IRB
IRB Office, Johns Hopkins Bloomberg School of Public Health
The Maryland Department of Health IRB
The University of Minnesota's Institutional Review Board
Minnesota Department of Health IRB
New York State Department of Health IRB
University of Rochester, Office for Human Subject Protection, Research Subjects Review Board
Oregon Health Authority's Public Health IRB

Supplementary Methods Appendix A. CALM Methodology

1. Introduction

The risk curve for anti-CPS IgG we aim to establish in this study is an example of a dose-response curve. We analyzed data from a natural immunity epidemiological study, which includes infants' antibody levels (exposure dose), their Group B Streptococcus (GBS) disease status (response), and potential confounding covariates.

When the response is binary, such as disease status (yes/no or 1/0), a dose-response curve is defined as a function of conditional probability:

$$f(t) = P(Y = 1 \mid T = t) \qquad [1]$$

where T represents the dose level, and Y denotes disease status. The curve is typically modeled parametrically based on the assumed underlying dose-response relationship, often using a monotonic function. A widely used parametric model is the two-parameter logit function, which exhibits a characteristic sigmoid shape:

$$f(t) = \frac{1}{1 + e^{\alpha+\beta t}} \qquad [2]$$

Other common parametric models are summarized in Daniels et al. [1].

However, real-world observational studies introduce challenges such as confounding variables and selection bias. Directly modeling the antibody-disease risk relationship without adjusting for these factors may lead to biased conclusions. Our proposed approach accounts for confounding effects and corrects for selection bias to ensure an accurate estimation of the antibody disease risk relationship.

2. Confounding Effects on Dose-Response Curves

To illustrate the impact of confounding variables on dose-response relationships, consider a single binary covariate X. Given observed data triplets $(y_i, t_i, x_i), i = 1, \ldots, n$, we first construct an overall dose-response curve, $f_{all}(t)$, using all pairs (y_i, t_i), ignoring the covariate. Next, we stratify by the covariate value and construct covariate-specific dose-response curves $f_1(t)$ and $f_0(t)$.

If $f_1(t) = f_0(t)$, then X does not affect disease risk at any antibody level and is not a confounder. In this case, we can estimate a common dose-response curve while ignoring X, since $f_{all}(t) = f_1(t) = f_0(t)$.

In general, if $f_1(t) \neq f_0(t)$, ignoring X may obscure meaningful biological differences between subgroups stratified by X. As shown in Panel A1 of Supplementary Figure 12, an overall dose-response curve may not adequately represent both subgroup-specific curves.

However, in a special case illustrated in Panel B1 of Supplementary Figure 12, although the covariate-specific dose response curves appear different, the relative effect of antibody remains invariant to the X. Panel B2 shows that when disease risk is scaled relative to the disease risk at the minimal antibody level t_0, the observed difference in Panel B1 disappear. By contrast, in panel A2, the difference between the two subgroups remains after the scaling, suggesting that the relative effect of antibody on disease risk remains depending on x.

The distinction is conceptually similar to the standard epidemiological approach for obtaining adjusted odds ratios in logistic regression. In logistic regression, odds ratios are also relative measures. If covariate-specific odds ratios vary significantly across subgroups, the covariate interacts with the exposure, making an overall odds ratio inappropriate. However, if covariate-specific odds ratios are similar, yet differ from the unadjusted odds ratio, the covariate is a

confounder. Logistic regression can adjust for its effect, yielding an adjusted odds ratio that better represents the common exposure effect across subgroups.

Applying this epidemiological reasoning from the context of an odds ratio based on a binary exposure to a dose-response curve based on a continuous exposure represents additional challenges. Our proposed method mirrors the standard epidemiological approach by first defining a relative measure that summarizes the antibody effect and then modeling the relationship between confounders and the relative antibody effect. Finally, we demonstrate that fitting the model to observed data correctly estimates the relative dose-response relationship free from confounding effects, and sampling bias.

Supplementary Figure 12. The absolute and relative covariate-specific dose response curve

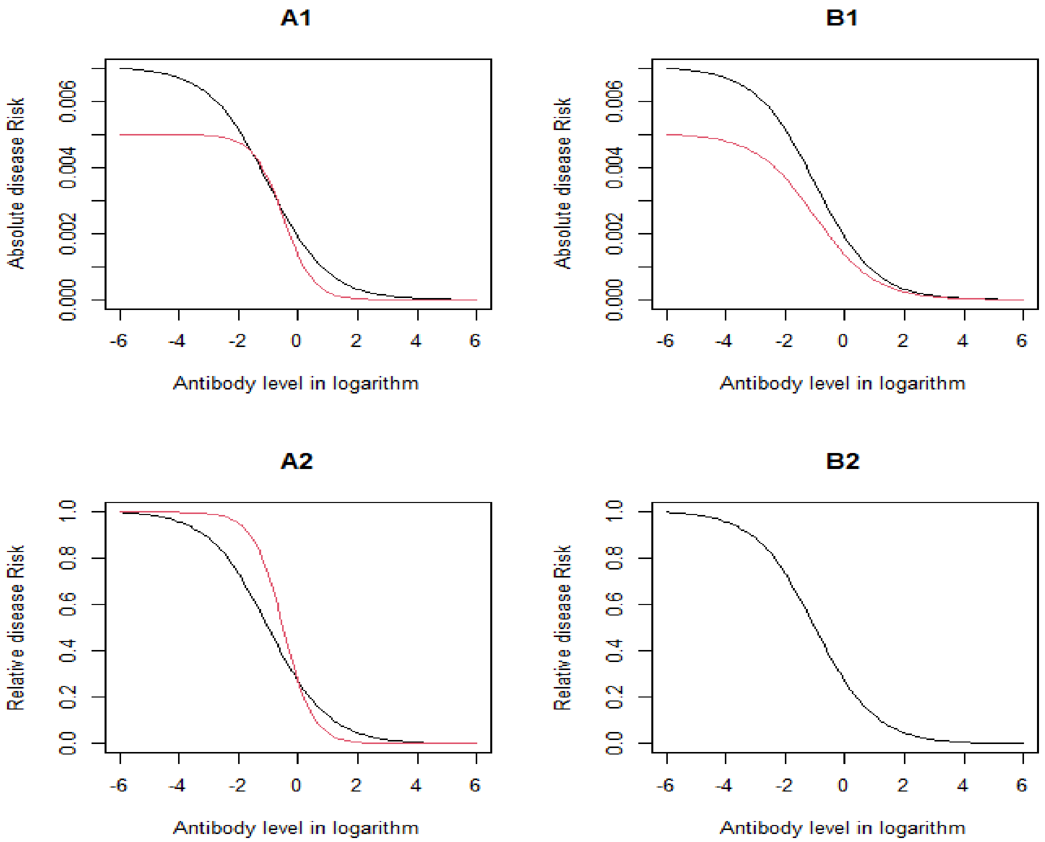

Legend: Panels A1 and B1 display absolute curves stratified by covariate x, where subgroup differences are evident. Panels A2 and B2 show the corresponding relative curves after scaling disease risk by the risk at the minimal antibody level (t0). Subgroup differences persist in A2, whereas in B2 the scaled curves coincide, indicating invariance to x.

3. Scaled Logit Model (SLM)

To improve the fit of antibody-disease data to the standard logit function (Equation [2]), Dunning [2] proposed the Scaled Logit Model (SLM) by introducing a scaling parameter λ,

$$f^s(t) = \lambda\, h(t) \qquad [4]$$

where $h(t)$ is a monotone function satisfying $h(t_0) = 1.0$ at the minimal dose level t_0. For example, $h(t)$ can be defined as: $h(t) = f(t)/f(t_0)$ where $f(t)$ is the standard logit function.

Beyond improving model fit, the SLM introduces two biologically meaningful quantities:

1. Baseline Disease Risk λ: Represents the disease risk at the minimal or zero antibody level, reflecting susceptibility to disease in the absence of antibody protection.

2. Relative Risk Reduction$h(t)$): Quantifies how disease risk decreases as antibody levels increase. For example, if $h(t_1) = 0.2$ at antibody level t_1, then disease risk decreases by 80% relative to the baseline risk at t_0.

Dunning also provided an alternative interpretation of $h(t)$ in the context of vaccine efficacy. Under an All-or-Nothing vaccine model -- where individuals are either fully immune or completely susceptible --- all subjects are susceptible at the minimal antibody level assuming no antibody protection at all. As antibody levels increase, the fraction of susceptible individuals declines according to $h(t)$.

The SLM framework, with its clear separation of baseline disease risk and relative risk reduction, forms the basis for our approach to adjusting dose-response curves for confounding factors and selection bias, analogous to logistic regression in traditional epidemiological analyses.

4. Covariate Adjusted Logit Model (CALM)

We extend the Scaled Logit Model (SLM) by incorporating covariate effects, defining the absolute dose-response function as:

$$f(t|x) = g(x)h(t) \qquad [5]$$

Here, the covariate-specific dose response curve is decomposed into:

1 Null Disease Risk (NDR) – the baseline disease risk now depends on covariate x, generalizing from the constant λ in SLM

2 Relative Dose-Response Curve $h(t)$ – a covariate-independent function quantifies the relative reduction in disease risk from the NDR, retaining its interpretation from SLM

We call our model Covariate Adjusted Logit Model (CALM). The model allows a common dose response relationship across different covariate values when the covariate is a confounder rather than interacting with antibody levels. While absolute disease risk may vary across subgroups (as seen in Panel B1) of Supplementary Figure 12, the relative reduction in risk remains consistent across strata. Thus, differences in disease risk among subgroups are captured by NDR $g(x)$, while $h(t)$ represents the universal antibody effect.

Parameter Estimation

If a parametric form is assumed for $g(x)$, (e.g., a logistic regression model), both $g(x)$, and $h(t)$ parameters can be estimated simultaneously using maximum likelihood estimation (MLE) by optimizing the log-likelihood function:

$$\log L = \sum_{i=1}^{n} [y_i \log\big(g(x_i)h(t_i)\big) + (1 - y_i) \log(1 - g(x_i)h(t_i))]$$

Nonlinear MLE methods are applied to obtain parameter estimates, as well as the corresponding standard errors.

Protection Threshold

Once $h(t)$ is determined, a protection threshold t_α can be identified such that:

$$h(t_\alpha) = 1 - \alpha$$

for a predefined protection level α. Confidence intervals for t_α can also be derived. The computations are implemented in **R.**

Model Validation

In addition to applying MLE for parametric models, as we did in this IgG analysis, in the development of CALM we also applied Bayesian approaches to estimate model parameters. We also explored non-parametric approaches to model $h(t)$. Extensive simulations confirm that under the CALM model framework, the relative dose-response curve $h(t)$ can be accurately estimated across varying levels of confounding and selection bias. Full details on CALM methodology and simulation results are provided in our methodology manuscript (Shang et al)[3]

Supplementary Methods Appendix B. Population disease rate calculations

- For early-onset disease, we assume approximately 25% of delivering mothers were colonized (denominator) and that all early onset cases come from colonized mothers (4)
  - For the numerator: we use the early onset cases from 2020 ABCs: 102
  - For the denominator: we use 25% of the live births reported in ABCs: (463,543*0.25)
  - Disease rate = 102/(463,543*0.25) = 0.00088
- For late-onset disease, we assume approximately 25% of delivering mothers were colonized (denominator) and that half of the cases had a colonized mother (5)
  - For the numerator: we use 50% of the late onset cases from 2020 ABCs: 135*0.5
  - For the denominator: we use 25% of the live births reported in ABCs: (463,543*0.25)
  - Disease rate = (135*0.5)/(463,543*0.25) = 0.00058

Supplementary References

1. Daniels, Robert D, Gilbert, Stephen J, Kuppusamy, Senthilkumar Perumal, Kuempel, Eileen D, ParkRobert M, Pandalai, Sudah P, Smith, Randall J, Wheeler, Matthew W, Whittaker, Christine and Schulte, Paul A. (2020). NIOSH Practices in Occupational Risk Assessment.
2. Dunning, Andrew J. (2006). A model for immunological correlates of protection. *Statistics in medicine* **25**(9), 1485 1497.
3. Shang, N, Schrag, S, Kahn, R, and Rhodes, J. Covariate Adjusted Logit Model (CALM) for Generating Dose-Response Curves from Observational Data with Applications to Vaccine Effectiveness Trials, MS ID#: MEDRXIV/2024/319273
4. Van Dyke MK, Phares CR, Lynfield R, Thomas AR, Arnold KE, Craig AS, et al. Evaluation of universal antenatal screening for group B streptococcus. N Engl J Med. 2009;360(25):2626-36.
5. Jordan HT, Farley MM, Craig A, Mohle-Boetani J, Harrison LH, Petit S, et al. Revisiting the need for vaccine prevention of late-onset neonatal group B streptococcal disease: a multistate, population-based analysis. Pediatr Infect Dis J. 2008;27(12):1057-64.
