# Supplementary material for: A US case-control study to estimate infant group B streptococcal disease serological thresholds of risk-reduction
Source: Nat Commun. 2025 Oct 23;16:9381. doi: 10.1038/s41467-025-64324-y (PMC12549997; doi:10.1038/s41467-025-64324-y)
Supplement: Supplementary file 2 — Reporting Summary [file 41467_2025_64324_MOESM2_ESM.pdf]

nature portfolio

Corresponding author(s): Rebecca Kahn

Last updated by author(s): Aug 18, 2025

Reporting Summary

Nature Portfolio wishes to improve the reproducibility of the work that we publish. This form provides structure for consistency and transparency in reporting. For further information on Nature Portfolio policies, see our Editorial Policies and the Editorial Policy Checklist.

Statistics

For all statistical analyses, confirm that the following items are present in the figure legend, table legend, main text, or Methods section.

n/a	Confirmed	
☐	☒	The exact sample size (*n*) for each experimental group/condition, given as a discrete number and unit of measurement
☒	☐	A statement on whether measurements were taken from distinct samples or whether the same sample was measured repeatedly
☐	☒	The statistical test(s) used AND whether they are one- or two-sided<br>*Only common tests should be described solely by name; describe more complex techniques in the Methods section.*
☐	☒	A description of all covariates tested
☐	☒	A description of any assumptions or corrections, such as tests of normality and adjustment for multiple comparisons
☐	☒	A full description of the statistical parameters including central tendency (e.g. means) or other basic estimates (e.g. regression coefficient) AND variation (e.g. standard deviation) or associated estimates of uncertainty (e.g. confidence intervals)
☐	☒	For null hypothesis testing, the test statistic (e.g. *F*, *t*, *r*) with confidence intervals, effect sizes, degrees of freedom and *P* value noted<br>*Give P values as exact values whenever suitable.*
☒	☐	For Bayesian analysis, information on the choice of priors and Markov chain Monte Carlo settings
☒	☐	For hierarchical and complex designs, identification of the appropriate level for tests and full reporting of outcomes
☒	☐	Estimates of effect sizes (e.g. Cohen's *d*, Pearson's *r*), indicating how they were calculated

Our web collection on statistics for biologists contains articles on many of the points above.

Software and code

Policy information about availability of computer code

Data collection	Data collection was conducted through the ABCs platform. More details here: https://www.cdc.gov/abcs/methodology/data-collection-and-forms.html.
Data analysis	Code is available as a supplemental file.

For manuscripts utilizing custom algorithms or software that are central to the research but not yet described in published literature, software must be made available to editors and reviewers. We strongly encourage code deposition in a community repository (e.g. GitHub). See the Nature Portfolio guidelines for submitting code & software for further information.

Data

Policy information about availability of data

All manuscripts must include a data availability statement. This statement should provide the following information, where applicable:

- Accession codes, unique identifiers, or web links for publicly available datasets
- A description of any restrictions on data availability
- For clinical datasets or third party data, please ensure that the statement adheres to our policy

Data can be shared confidentially with editors and peer reviewers upon request for the sole purpose of refereeing the manuscript. In addition, we have added the following statement to the manuscript:

'The data that support the findings of this study are not openly available to protect the privacy of the study subjects. Study data can be made available upon

request, including completion of Data Use Agreement. Data requestors can contact the corresponding author directly or submit a request here: https://www.cdc.gov/abcs/isolates-and-data/isolate-and-data-requests.html'

Research involving human participants, their data, or biological material

Policy information about studies with human participants or human data. See also policy information about sex, gender (identity/presentation), and sexual orientation and race, ethnicity and racism.

Reporting on sex and gender	Infant sex was abstracted from the infant's medical chart and is reported in Table 1 as a characteristic of the study population. The study findings are not sex-specific. Sex- and gender-based analyses were not performed as neither were identified as potential confounders in our directed acyclic graph.
Reporting on race, ethnicity, or other socially relevant groupings	Infant race and ethnicity was abstracted from the infant's medical chart and is reported in Table 1 as a characteristic of the study population. The race and ethnicity categories collected reflect minimum categories and definitions recommended by the Office of Management and Budget-15 Directive (OMB-15) for federal agencies during the study period. The study findings are not race- or ethnicity-specific and the impact of race/ethnicity was explored through a sensitivity analysis - see supplemental Figure 6.
Population characteristics	Our study population consisted of case and control infants <90 days old. Select maternal, intrapartum, and newborn characteristics are described in Table 1.
Recruitment	Cases were identified through CDC's Active Bacterial Core surveillance (ABCs). Controls were identified by clinical center partners performing routine antenatal and intrapartum Group B Streptococcus screening. Cases and controls were enrolled by individual-level parental consent when required, or through waivers of individual-level consent and de-identification when this alternate approach was approved. Additional details are provided in Figure 1. Study population enrollment flow diagram.
Ethics oversight	This study was reviewed and approved by CDC and site-level Institutional Review Boards (IRBs) listed in Supplemental Table 1.

Note that full information on the approval of the study protocol must also be provided in the manuscript.

Field-specific reporting

Please select the one below that is the best fit for your research. If you are not sure, read the appropriate sections before making your selection.

[x] Life sciences [ ] Behavioural & social sciences [ ] Ecological, evolutionary & environmental sciences

For a reference copy of the document with all sections, see nature.com/documents/nr-reporting-summary-flat.pdf

Life sciences study design

All studies must disclose on these points even when the disclosure is negative.

Sample size	Sample size was determined based on feasibility (time to enroll based on incidence of infant GBS disease in the US, and the urgent need for evidence regarding immunologic endpoints) combined with a desire for as precise estimates as possible.
Data exclusions	Figure 1 provides a detailed flow chart of study enrollment and all exclusions applied.
Replication	N/A
Randomization	N/A
Blinding	N/A

Reporting for specific materials, systems and methods

We require information from authors about some types of materials, experimental systems and methods used in many studies. Here, indicate whether each material, system or method listed is relevant to your study. If you are not sure if a list item applies to your research, read the appropriate section before selecting a response.

Materials & experimental systems

n/a	Involved in the study
☒	☐ Antibodies
☒	☐ Eukaryotic cell lines
☒	☐ Palaeontology and archaeology
☒	☐ Animals and other organisms
☒	☐ Clinical data
☒	☐ Dual use research of concern
☒	☐ Plants

Methods

n/a	Involved in the study
☒	☐ ChIP-seq
☒	☐ Flow cytometry
☒	☐ MRI-based neuroimaging

Plants

Seed stocks	*Report on the source of all seed stocks or other plant material used. If applicable, state the seed stock centre and catalogue number. If plant specimens were collected from the field, describe the collection location, date and sampling procedures.*
Novel plant genotypes	*Describe the methods by which all novel plant genotypes were produced. This includes those generated by transgenic approaches, gene editing, chemical/radiation-based mutagenesis and hybridization. For transgenic lines, describe the transformation method, the number of independent lines analyzed and the generation upon which experiments were performed. For gene-edited lines, describe the editor used, the endogenous sequence targeted for editing, the targeting guide RNA sequence (if applicable) and how the editor was applied.*
Authentication	*Describe any authentication procedures for each seed stock used or novel genotype generated. Describe any experiments used to assess the effect of a mutation and, where applicable, how potential secondary effects (e.g. second site T-DNA insertions, mosiacism, off-target gene editing) were examined.*
